# Virulence of recurrent infestations with *Borrelia*-infected ticks in a *Borrelia*-amplifying bird

**DOI:** 10.1038/srep16150

**Published:** 2015-11-10

**Authors:** Dieter J. A. Heylen, Wendt Müller, Anke Vermeulen, Hein Sprong, Erik Matthysen

**Affiliations:** 1 Evolutionary Ecology Group, Department of Biology, University of Antwerp, Belgium; 2 Ethology Group, Department of Biology, University of Antwerp, Belgium; 3 Laboratory for Zoonoses and Environmental Microbiology, National Institute for Public Health and Environment (RIVM), Bilthoven, the Netherlands

## Abstract

Lyme disease cases caused by *Borrelia burgdorferi* s.l. bacteria is increasing steadily in Europe, in part due to the expansion of the vector, *Ixodes ricinus*. Wild reservoir hosts are typically recurrently infested. Understanding the impact of these cumulative parasite exposures on the host’s health is, therefore, central to predict the distribution of tick populations and their pathogens. Here, we have experimentally investigated the symptoms of disease caused by recurrent infestations in a common songbird (*Parus major*). Birds were exposed three times in succession to ticks collected in a *Borrelia* endemic area. Health and immune measures were analyzed in order to investigate changes in response to tick infestation and *Borrelia* infection rate. Nitric oxide levels increased with the *Borrelia* infection rate, but this effect was increasingly counteracted by mounting tick infestation rates. Tick infestations equally reduced haematocrit during each cycle. But birds overcompensated in their response to tick feeding, having higher haematocrit values during tick-free periods depending on the number of ticks they had been previously exposed to. Body condition showed a similar overshooting response in function of the severity of the *Borrelia* infection. The observed overcompensation increases the bird’s energetic needs, which may result in an increase in transmission events.

Transmission and amplification of vector-borne diseases depend among other things on the constraints imposed by the host on both the pathogenic agent and its vector. This is because natural selection leads to the evolution of strategies that limit ‘virulence’ of parasites in hosts, i.e. their capacity to decrease some component of host fitness^1^. Those physiological^2^ and behavioural^3^ constraints reduce, compensate or avoid detrimental effects on the host, but at the same time they impact on the population dynamics of the parasites. Mechanisms of disease that select for these strategies in the host, are at the proximate base of host–parasite interactions. It is, therefore, crucial to interpret the health impact caused by the parasites as an outcome of the co-evolution between parasites and hosts. Parasitic vectors of diseases are particularly interesting in this context, since they challenge the host twofold. The vectors themselves tend to drain resources from their hosts and/or they cause pathological effects^4^,^5^,^6^. But they are also known to facilitate pathogen transmission, often by modulating the host’s immune response^7^,^8^,^9^. The vector-borne parasites may, in turn, induce an additional reduction in host survival and reproductive success by causing disease. Although vector and transmitted pathogens exploit the same host individual, the temporal dynamics of their effects may differ. Hosts can become chronically infected by pathogens, and may develop systemic infections over a long period of time^7^,^10^,^11^. The vector, in turn, often exploits the host for much shorter periods of time while provoking acute effects. However, while it is in most cases unlikely that a vector individual re-infests the same host individual, hosts can be repeatedly exploited by the same type of parasitic vectors. The effects of recurrent infestations and infections are likely to impinge on each other, and under some circumstances vectors may also provoke chronic symptoms of disease, in addition to their immediate acute effects^12^. Yet to date, little is known on the extent to which vectors and their pathogens jointly affect the physiological health status of their natural wildlife hosts, both in a chronic and an acute way.

One such vector-pathogen system that enables us to address these questions consists of the blood-sucking tick, *Ixodes ricinus*, and the tick-borne *Borrelia burgdorferi s.l. bacteria (hereafter Borrelia). Ixodes ricinus* is the main vector of the *Borrelia* bacteria in Europe, that infests a wide range of terrestrial vertebrate host species, including birds, reptiles and mammals. This tick has a three-stage life cycle (larva, nymph, and adult), and it feeds once during each developmental stage^13^. As the uninfected ticks feed on an infected host tissue, the *Borrelia* spirochetes are taken up in the blood meal^14^. It is recognized that birds make a significant contribution to the cycles of *Borrelia* bacteria^10^,^15^,^16^. Aside from carrying and spreading pathogen-infected ticks, some bird species are able to sustain tick-borne disease infections in their body for long time periods, and they may, therefore, act as reservoir hosts that transmit diseases to uninfected ticks^10^,^16^. In the holarctic region, the highest abundances of questing ticks (April–July) coincides with the season when birds rear their nestlings and, more specifically, with the moment when nestlings fledge^13^,^17^,^18^,^19^. At this time of the year, songbirds that forage inside the habitat of *I. ricinus* (i.e. the lower vegetation strata) are repeatedly exposed to and infested with *Borrelia* infected ticks. Juvenile birds are of particular interest as they have a low acquired immunity, and thereby may enhance the maintenance of tick-borne pathogens in endemic areas due to a reduced resistance against tick feeding, and by facilitating the proliferation and transmission of these pathogens^20^,^21^,^22^. Nevertheless, parasite infections in hosts with low acquired immunity may result in adverse health effects that affect the birds’ survival and reproduction, and hence could ultimately affect the maintenance and spatial distribution of the ticks and their pathogens in the wild.

Here, we experimentally study (1) the effects of repeated exposure to the *Ixodes ricinus* -*Borrelia* system on a number of health parameters in a common bird species, the great tit (*Parus major* L.), (2) whether these effects relate to the tick loads, the degree of infection with the tick-borne pathogen, or both and (3) whether the acute infestation effects depend on the degree of previous infestations with ticks and/or *Borrelia*.

We used the great tit (*Parus major* L.) as a tick host, as this bird is among the most abundant resident birds of European woodlands and gardens, and is frequently infested by *Ixodes ricinus* ticks that are themselves infected by a variety of *Borrelia* genospecies^23^,^24^. Naïve juvenile birds that had not been previously exposed to *Borrelia* were artificially infested with ticks during three successive sessions, each lasting 4–5 days (the normal time for ticks to engorge), punctuated with periods of 5–6 days without ticks (Fig. 1). The effects on the health status of the host were measured via changes in body condition, haematocrit levels, inflammation and immune measures in the blood.

## Results

The number of ticks that successfully fed on the birds (Inf. 1 + Inf. 2) varied among individuals, ranging from 15 to 34 ticks per bird (Figs 2 and 3). The average number of ticks that successfully fed during each infestation session was: 13.4 ± 0.6, 12.7 ± 0.8, and 11.4 ± 0.8 per bird for Inf. 1 to Inf. 3, respectively. The infestation success did not significantly change with infestation order^20^. *Borrelia* DNA was detected in 12.2% of the 1414 unfed ticks. The average infection rate (i.e. the proportion of ticks being infected per host) significantly increased with each infestation cycle^22^, being 40 ± 4%, 73 ± 5%, 88 ± 4% for Inf. 1 to Inf. 3, respectively. There was considerable variation among individuals, with cumulative infection rates (sum Inf. 1–3) per bird ranging from 21 to 86% (Figs 2 and 3). The bird-specific *B. garinii* dominated the *Borrelia* community, comprising more than 80% of the genotyped tick extracts^22^. The *Borrelia* infection rates were not significantly correlated with tick infestation success (Pearson’s rho: −0.1; P = 0.64). None of the health parameters measured immediately before tick exposure 1 (Inf. 1) were associated with the feeding success of the ticks, or *Borrelia* infection rates (all P’s > 0.05).

During the experiment, five birds died (one, two and two during sessions 1, 2, and 3, respectively). One bird that died in session 1, had a high infection rate of 87% (in 16 fed ticks), which was the highest in rank for all birds. All other birds that died had *Borrelia* infection rates (range rank orders 12–20) and infestation loads (range rank orders 12–22) that were similar to those birds that survived.

### Cumulative health impact of recurrent exposures

Parameter estimates of the models that test for the change in health parameter (Δ Inf. 3–1) in dependence of the *Borrelia* infection rate and the number of ticks that fed on the birds are presented in Table 1.

We found that the increase in body condition over the infestation sessions was higher in birds that had more *Borrelia* infected ticks (interaction session x *Borrelia*: 0.055 ± 0.015 g/mm; F_1,27.1_ = 13.2, P = 0.0012; Fig. 2). Furthermore, the Hct levels significantly changed throughout the observation period as a function of tick loads, with a stronger increase in Hct in birds that carried more ticks (session x ticks = 0.0015 ± 0.0006; F_1,37.4_ = 6.85, P = 0.012). For the NOx levels, we found an interactive effect of tick exposure and *Borrelia* infection rates: The change in NOx levels decreased with tick load (session x ticks = −0.0011 ± 0.0004 mmol/L; F_1,46_ = 5.62, P = 0.02), but the higher the *Borrelia* infection rates, the more the NOx concentration increased (session x ticks x *Borrelia* = 0.0016 ± 0.0007 mmol/L; F_1,46_ = 4.9, P = 0.03). In none of the other immune measures was the change with infestation session related with the *Borrelia* infection rates or tick loads. Agranulocyte numbers increased over the course of the experiment (log-link: 0.43 ± 0.09/10,000; F_1,47_ = 21.0, P < 0.0001), but this was not related to the parasites.

### Changes in acute effects between infestation 1 and 3

**Figure 1:**
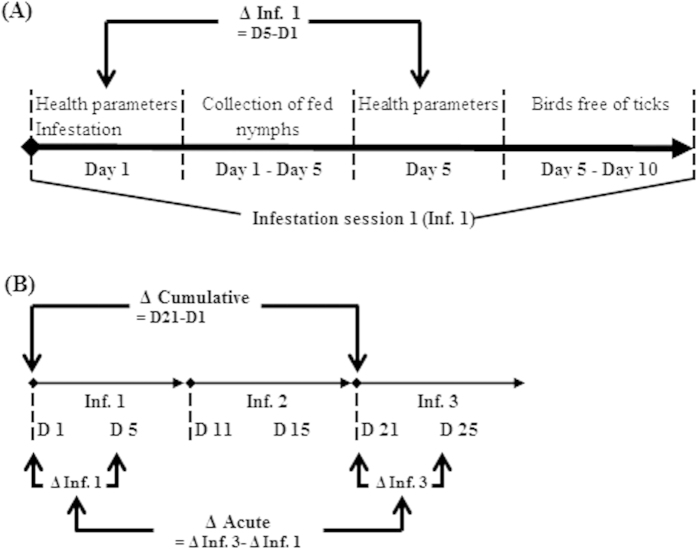
Schematic overview of the study design. (**A**) Actions taken during an infestation session (Inf.). “Δ Inf. 1” denotes the acute effect of the tick exposure (17 nymphs) on the birds’ physiology in session 1, i.e. the change in physiological parameters from prior to infestation to the moment that all ticks detached. (**B**) Schedule of successive infestation sessions. “Δ Cumulative” denotes the change in physiological parameters when birds had the opportunity to recover from the acute tick effects of Inf. 1 and Inf. 2 compared to the initial value in at day 1 (D1). “Δ Acute” denotes the change in acute responses between Inf. 1 and Inf. 3.

Model parameter estimates for the change in acute effects of the tick exposure (Δ Inf. 3 minus Δ Inf. 1; see Fig. 1) in relation to the *Borrelia* infection rate and the total number of ticks are presented in Table 2.

Hct levels decreased during each tick infestation (−0.0012 ± 0.0003; F_1,48_ = 13.01, P = 0.0007), but this acute effect did not differ between infestation 1 and 3. The acute effect of the ticks on ESR decreased significantly over the infestation sessions (Δ Inf. x session: −0.003 ± 0.001; F_1,47_ = 9.09, P = 0.004), but there was no statistical association with any of the parasites. The acute NOx responses were higher in Inf. 3, but this increase was counteracted by the negative correlation between NOx and *Borrelia* infection rate (Δ Inf. x session x *Borrelia*: −0.0010 ± 0.0002; F_1,39.3_ = 12.32, P = 0.001). No other significant effects were found in any of the other health characteristics studied.

## Discussion

The present study is the first to experimentally investigate both cummulative and acute health effects of a parasitic vector and its vector-transmitted pathogens in a wild bird species. Our experiment mimics the natural situation, whereby songbirds are recurrently exposed to *Ixodes* ticks and their endemic *Borrelia* strains. We focused on the juvenile post-fledging stage, which is the time when tick-borne diseases are more likely to be established for the first time. This is in contrast to most previous studies which investigated the occurrence of tick infestations and *Borrelia* infections at the time of autumn and spring migration^10^,^24^,^25^,^26^. This life history stage is of particular interest, due to a low acquired resistance, but also because of the high propensity to disperse and establish new territories^27^ that may lead to the spatial expansion of tick populations and their transmitted pathogens.

### Effects of tick parasitism

**Figure 2:**
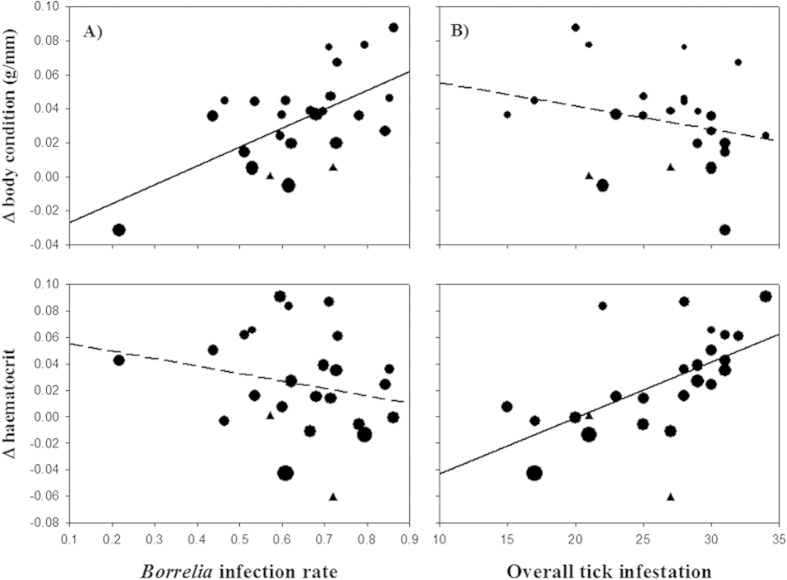
Changes in the health measures (Δ Cumulative, see Fig. 1) of great tits in relation to the *Borrelia burgdorferi*s.l. infection rate (A) and overall tick exposure (B). Least squares regression lines are fitted. Slope differs statistically significantly from zero (solid line) or not (dashed lines). The size of the symbols portrays the actual difference in the acute response between infestation 3 and 1 (Δ Acute, see Fig. 1). Triangles represent the birds that died during infestation 3.

Birds that died did not have particularly high tick loads, except for one bird. This suggests that *I. ricinus* infestations are not associated with (short-term) survival. However, all birds suffered each time from acute blood depletions caused by feeding ticks resulting in reduced haematocrit levels - independent of previous tick exposures^20^,^28^,^29^. This supports the hypothesis of a lack of resistance to ticks in this songbird species^20^. Birds succeeded to compensate, and even over-compensated for the erythrocyte loss. This increase was stronger when the previous tick burdens were higher (Fig. 2). The latter could be explained by a density-dependent stimulation of host erythropoiesis by molecules secreted by ticks^30^ or via erythrocyte decreases^31^. Interestingly, the leukocyte counts did not change with tick loads. In vertebrate host species that acquire resistance to ticks, these measures increase when exposed^8^. But in our study species, tick infestation success remained high^20^, indicating that the ticks (still) succeeded in circumventing and/or suppressing the birds’ haemostatic processes, inflammatory responses, and the immune counterattacks for preventing blood loss^8^,^9^. A similar observation in which birds tolerate repeated tick exposures, without developing resistance, has been observed in *Turdus migratorius*^16^.

Despite the energy losses linked to the tick’s repeated erythrocyte drainage, and the bird’s compensatory processes, the general body condition did not decrease. Instead, in most of the birds, the body condition increased throughout the experiment (Fig. 2) which was possibly facilitated by the *ad libitum* food conditions. Even though we performed our experiment in a series of consecutive blocks that started within a short time window (20 days), we cannot fully exclude that seasonal effects play a role here too. The observed increase may also relate to the gain in body mass for the regeneration of feathers (i.e. the post-juvenile moult)^32^. Similarly, seasonal changes in agranulocytes have been reported for other wild bird species, which has been interpreted as an indication of a shift toward lymphocyte-based immunity^33^,^34^. Such temporal or developmental processes have, therefore, to be kept in mind when interpreting the variation in part of the acute response to tick infestation (e.g. ESR, Table 2).

But our experimental manipulation only explores the consequences of interrupted recurrent infestations, while infestations may follow each other more rapidly or even overlap under natural conditions. Thus our results are the outcome of one possible scenario, and it requires additional studies to investigate changes in tick virulence throughout a broader range of scenarios.

### Effects of *Borrelia* infection and their interaction with tick parasitism

**Figure 3:**
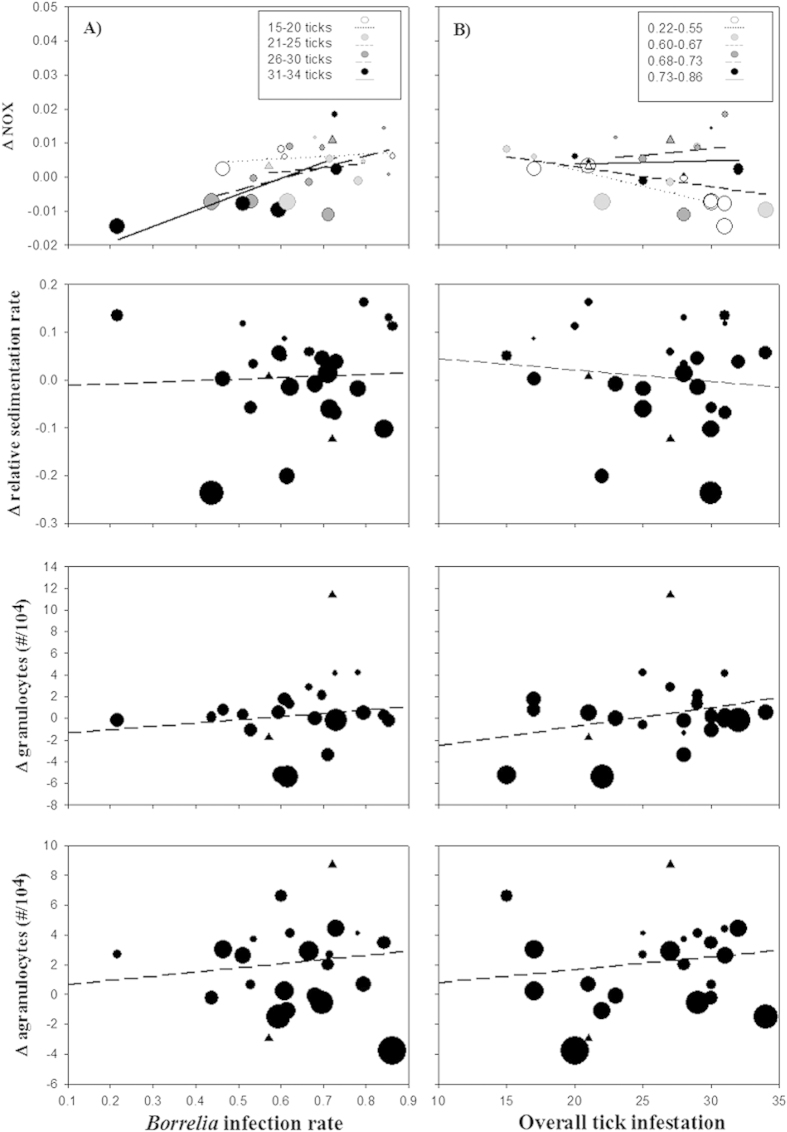
Changes in immune measures (Δ Cumulative, see Fig. 1) of great tits in relation to the *Borrelia burgdorferi*s.l. infection rate (A) and overall tick exposure (B). Least squares regression lines are fitted. Except for the presented Tick x *Borrelia* interaction in NOx, slopes that differ statistically significantly from zero are indicated by a solid line. The size of the symbols portrays the actual difference in the acute response between infestation 3 and 1 (Δ Acute, see Fig. 1). Triangles represent the birds that died during infestation 3.

In addition to the above mentioned seasonal changes in among others body condition, we found that this increase correlated with the *Borrelia* infection rate. This is highly interesting, since such an increase contrasts with the substantial nutritional and energetic costs that are required to trigger and maintain an inflammatory response^35^,^36^. Birds may possibly anticipate the costs of an activation of the immune system due to ongoing, non-cleared *Borrelia* infections. From the pathogen perspective, *Borrelia* spirochetes could proliferate more successfully in those birds with a stronger body mass increase and it is conceivable that they may even induce such metabolic processes (cf. *Borrelia* stimulating fat decompositions in *I. ricinus*^37^). When considering the effects of a *Borrelia* infection on the songbird’s immune system, we found an interactive effect on the NOx levels between *Borrelia* infection and the tick loads during previous infestations. NOx has signaling functions in the cascade of an immune response and may also act as a direct non-specific cytotoxin^38^,^39^. Here, NOx levels decreased with tick infestation levels, but this change was counteracted by the *Borrelia* infection rate (Fig. 3). The decrease of NOx with tick loads can most likely be explained by the immuno-suppressive effects of molecules secreted by the tick saliva^40^,^41^. This may ultimately also facilitate the proliferation of vector-borne diseases^42^. A stimulation of NOx production by *Borrelia* infections as shown here, has also been observed in mammals^43^,^44^, where the signal function of NOx plays a significant role in the processes that aim at eliminating *Borrelia* spirochetes via macrophages^45^. Interestingly, the acute NOx response against the final tick exposure was lower when the previous *Borrelia* infection rate (and hence NOx level) was already high (Fig. 3). This association suggests a physiological upper limit in the immune reaction, possibly due to a high immune-pathological cost. It could however also indicate that those birds that had already elevated NOx levels because of an ongoing *Borrelia* infection, were more effective in eradicating additionally introduced bacteria of infestation 3, and hence show a smaller NOx increase. The outcome may also suggest an acquired immune response against *Borrelia*, which would probably be associated with the downregulation of effectors such as NOx. Identifying the specific immunological pathways in birds against tick-*Borrelia* exposures requires further research. The NOx association in great tits indicates that competent reservoirs, which are expected to carry infections without showing signs of disease^46^, may still present measurable symptoms of infection. Nevertheless, the effects of the bacteria on health and mortality in great tits seem to be low, which is in line with findings in other infected native^10^,^16^,^47^,^48^ and domesticated birds^49^,^50^.

### Implications for tick exposures and *Borrelia* transmissions

Pathogen infections may alter host physiology and behaviour in a way that positively influences transmission^51^,^52^,^53^, and compensatory processes such as those observed in this study may belong to this category. They probably increase the nutritional needs, hence foraging efforts, that ultimately enhance the encounter rates with uninfected ticks, hereby increasing the transmission and reproduction of the *Borrelia* spirochetes. This effect may become reinforced if the higher demand pushes the juveniles to forage in sub-optimal habitat, the lower vegetation strata, entailing a higher tick infestation risk. Indeed, brood-rearing great tits had more severe tick burdens when raising larger broods with higher food requirements^19^. On the other hand, the higher haematocrit levels (reflecting elevated capacity of oxygen consumption) and body mass (reflecting higher energy reserves), both enable hosts to be more active, have larger home ranges and possibly to investigate a greater proportion of the space within their home range, again increasing the chances of encountering ticks^54^,^55^. Studying whether and to what extent the observed physiological effects alter the bird’s susceptibility to future tick infestations seems, therefore, to be a promising line for future research.

The present study contributes to our understanding of the virulence of the parasitic actors in a common tick-pathogen system in a native resident songbird reservoir. The outcome can be regarded as an indication of tolerance rather than resistance to the vector and its vector-borne disease, with tolerance being defined as mechanisms reducing the impact of the parasite on the host rather than preventing the exploitation by and proliferation of the parasites^56^. Repeated parasite exposures led to an overcompensation in health measures, which in the end may benefit the fitness of both ticks and *Borrelia* spirochetes. Furthermore, repeated tick exposures not only increase the *Borrelia* transmission occasions, but while attached to the bird, ticks also suppress the bird’s immune response, potentially enhancing the *Borrelia* proliferations.

Thus, taken together juvenile great tits act as potent *Borrelia* reservoirs during the period of post-fledging dispersal, with subtle clinical symptoms of infections. This probably facilitates subsequent transmission, and will have a significant impact on the spatial distribution of *Borrelia* and its vector. As a next step, it needs to be shown whether similar effects can be observed for adult birds with maturated immune systems and other species. Furthermore, infestations may often overlap or may cover longer time periods. The consequences of these patterns have, as yet, to be studied.

## Methods

### Birds and ticks

The great tit is a small passerine bird (body mass 15–20 g) which commonly breeds in woodlands and gardens throughout the Palearctic. Great tits act as competent reservoir for *Borrelia*^22^,^25^,^57^,^58^ and juvenile birds do not acquire resistance to infestations with the most important European vector, *Ixodes ricinus* nymphs^20^. Wild *Borrelia*-free birds were obtained a few days before fledging by collecting nestlings from parasite-free, artificial nest boxes, which great tits readily accept for breeding^59^,^60^ (under licence N° S8/VERG/07-U5R26 of the Agency for Nature and Forests, Flemish Government, Belgium) (for details see^20^). They were introduced together with their parents into tick-free outdoor aviaries located at the University of Antwerp (Belgium), where the parents continued to feed the nestlings until independence. In total 28 naïve *P. major* individuals were used in the experiment. Birds received water and food *ad libitum* during their stay in captivity, and had the opportunity to take a bath in fresh water. All procedures, including the tick infestation, were carried out in accordance with national environmental legislation and regulations, and were approved by the Ethical Committee for Animal Experiments of the University of Antwerp (License N° 2009-32).

*Ixodes ricinus* nymphs (approximately 3000) were caught by dragging a white flannel flag over suitable vegetation in an area that is endemic for *Borrelia*. The ticks were subsequently kept under sterile conditions in a climate room at >90% relative humidity, a 16 h:8 h (light:dark) photoperiod, and a 25 °C:15 °C temperature cycle until infestation. After feeding on *P. major*, the engorged nymphs were kept in individual tubes at 25 °C and >90% relative humidity^61^ until moult to the adult stage was completed, after which ticks were screened for *Borrelia*.

### Study design

When the birds were 9 weeks old, individuals were infested with *I. ricinus* nymphs three times in succession (Infestation 1–3) (see Fig. 1). Each infestation lasted 4–5 days, and the birds were kept free of ticks for a duration of 5–6 days between the consecutive infestations. Within 20 days, three batches were involved in the experiments (9–10 birds per batch). Batches of birds were stratified according to nest of origin. We infested birds with tick loads corresponding to the maximum level found under natural conditions in our study population. To this end, 17 randomly selected *I. ricinus* nymphs were put underneath the feathers on the head of each bird in each infestation session using moistened tweezers. Birds were then kept for 2 h in an air-permeable cotton bag (size: 20 cm × 15 cm) inside a darkened cage which kept them inactive^28^. After tick exposure, birds were placed in individual cages with a wire-mesh floor (40 cm × 80 cm). Below the wire-mesh was a plastic tray containing damp filter paper and edges were streaked with vaseline to prevent nymphs from escaping. The engorged nymphs that dropped through the mesh cage were collected each day with minimal disturbance to the host.

We measured the effects of the *Borrelia* infection and the cumulative exposure to ticks (Inf. 1+ Inf. 2) on health measures as the change in health status between the moments immediately before tick exposure 1 and immediately before tick exposure 3 (i.e. 5–6 days after Inf. 2). *Borrelia* infection rate in the ticks are obtained from the three infestation sessions, that is the proportion of ticks that were *Borrelia* positive. They were used as a proxy for the bird’s *Borrelia* infection status built up during the first 20 days (hereafter: ‘*Borrelia* infection rate’). This time period is assumed to be sufficiently long to measure the degree of systemic infectivity, based on literature data on the latent period between the hosts being bitten by an infected tick and becoming infective (6–12 days in American robins *Turdus migratorius*^16^, 14 days in juvenile chickens *Gallus gallus*^62^).

Furthermore, we studied the change in the acute responses (i.e. the change in health status immediately before and just after tick exposure; Fig. 1A) between infestation 1 and infestation 3 (Fig. 1B) in response to the cumulative exposure to the ticks and the *Borrelia* infection status.

### Health measures

We measured six parameters reflecting the hosts’ health status immediately before and after the infestation (Fig. 1A). (1) Body condition (mass/tarsus ratio): Body mass was measured to the nearest 0.1 g using a digital balance. We subsequently calculated the ratio between body mass and a skeletal measurement (tarsus length, measured with a digital caliper to the nearest 0.01 mm) as a measure of body condition e.g.^63^,^64^. All other health parameters were measured from a blood sample (maximum 65 μL) that was taken from the ulnar vein collected into 75 μL heparinized capillary tubes. (2) Erythrocyte sedimentation rate (ESR): The ESR is a diagnostic method based on the fact that the movement of erythrocytes through plasma is enhanced by increased levels of major acute-phase proteins and immunoglobulins. High values signify an impaired health status^65^,^66^. To measure ESR, heparinized capillary tubes containing blood samples were placed vertically for 4 h at 4 °C. The level of ESR was measured by calculating the ratio between the volume of the part of a capillary tube not occupied by red blood cells and the total volume of blood in the capillary tube. As this measure is correlated with the haematocrit level (Hct) (Pearson’s rho = −0.32; P < 0.0001), for further statistical analyses we calculated its residuals of a least squares regression with Hct. (3) Haematocrit level: After measuring the ESR, blood samples were centrifuged for 10 min at 14,000 *g* to measure the Hct. Anaemia, as indicated by low Hct, results in a reduced oxygen-carrying capacity of the blood and consequently restricts oxygen-demanding processes^67^. ESR and Hct were measured with a digital calliper to the nearest 0.01 mm under optimal light conditions. (4) Agranulocyte and (5) granulocyte counts (#/10,000 erythrocytes): A drop of blood was smeared on a marked microscope slide, air-dried, fixed in 100% methanol, stained according to a 3-step Romanovsky stains procedure and scanned for 15 min under 400x and 1,000x magnification with oil immersion. None of the blood smears showed infestations with protozoan blood parasites. Before scanning for hematozoa, slides were used to measure leukocyte concentrations (expressed as the number of leukocytes per 10,000 erythrocytes). The total number of leukocytes among approximately 40,000 erythrocytes under 400x magnification were counted. Leukocyte concentrations obtained by this method have been shown to be repeatable^28^. (6) Nitric oxide concentrations (NOx): Plasma samples were stored at −80 °C until analysis of NOx (mmol/L). Nitric oxide acts as an immune effector contributing to the control of the acute phase of infection, and NOx measurements can be considered as a robust tool for the assessment of the magnitude of innate immune response and pathogenicity of infections^38^,^68^. We used a spectrophotometric assay based on the reduction of nitrate to nitrite by copper-coated cadmium granules, followed by colour development with a Griess Reagent System according to the protocol described in^68^.

### PCR-based detection of Borrelia in ticks

1414 unfed nymphs and 854 nymphs that had fed and moulted to the adult stage, were immersed in 70% ethanol and stored at −20 °C before testing. All the ticks were screened for the presence of *Borrelia burgdorferi* s.l. DNA was extracted by alkaline lysis^69^. For the detection of *B. burgdorferi* s.l., a duplex qPCR was redesigned based on existing qPCR protocols on fragments of the OspA^70^ and flagellin^71^ genes. For a detailed description of primers, probes an qPCR protocol we refer to^23^. For each PCR and multiplex qPCR, positive, negative controls and blank samples were included. A 10^−3^ to 10^−5^ dilution of DNA from a *B. burgdorferi* culture (*B. burgdorferi* s.s. B31 strain) was used as a positive control. From all qPCR-positive tick lysates, *B. burgdorferi* s.l. genospecies were determined by PCR amplification and sequencing of the variable 5S-23S (rrfA-rrlB) intergenic spacer region. In order to minimize contamination, the PCR procedures were performed in three separate rooms, of which the reagent setup and sample addition rooms were kept at positive pressure, whereas the DNA extraction room was kept at negative pressure. All rooms had airlocks.

### Statistical analysis

GLMM’s (Generalized Linear Mixed Models) were fitted on the health measures ((a)granulocyte counts: poisson distributed residuals, all other variables: normally distributed residuals) to model the change in their initial values (Δ Inf. 3-1) (= Cumulative effects) as a function of the following main effects: total number of ticks (Inf. 1 + 2), *Borrelia* infection rate in the ticks and their higher order interactions. In a second analysis, we tested whether the changes in acute effects of tick infestation (‘Δ Acute’, Fig. 1) are related to the same main effects as above. For all analyses, we corrected for the bird’s sex. Only the baseline values (i.e. intercepts at day 0 of Inf. 1) differed between the sexes in two of the health measures: Hct (Δ _male-female_: 0.024 + 0.008; T_df=21.1_ = 3.1; P = 0.005) and condition (Δ _male-female_: 0.06 + 0.02 g/mm; T_df=21.9_ = 4.1; P < 0.001), for which we controlled in the models. None of the higher interaction terms with sex differed significantly from zero, and the results related to sex will, therefore, not be reported further.

We added a random intercept effect on the level of bird individual, consequently we took into account the correlation among individuals of the same cluster. In all models, a stepwise selection procedure was used in which the model was iteratively refitted after exclusion of the least significant effect, until only significant factors and their lower order interactions terms were left. For the inference of the maximum likelihood estimates of the fixed effects, Kenward Roger approximation was used to estimate the denominator degrees of freedom of the F-distributed test statistics, which takes into account the correlation of observations within the same cluster^72^,^73^, i.e. the bird individual. All data manipulations and statistical analyses were performed using SAS v 9.2 (SAS Institute, Cary, North Carolina, USA). Estimates are reported as mean ± standard error.

## Additional Information

**How to cite this article**: Heylen, D. J. A. *et al.* Virulence of recurrent infestations with *Borrelia*-infected ticks in a *Borrelia*-amplifying bird. *Sci. Rep.* **5**, 16150; doi: 10.1038/srep16150 (2015).

## Figures and Tables

**Table 1:** Cumulative health impact of recurrent exposures.

	Health measures		Immune measures			
	Body condition	Haematocrit	Sed. Rate	Nitric Oxide	Granulocyte	Agranulocyte
Fixed						
Session	F_1,26.9_ = 3.55	F_1,36.7_ = 3.06	F_1,49_ = 0.06	F_1,46_ = 3.49	F_1,47_ = 0.50	F_1,47_ = 21.0 ***
	−0.019 ± 0.010	−0.027 ± 0.015		0.02 ± 0.01		0.43 ± 0.09
Session * *Borrelia*	F_1,27.1_ = 13.2 **	F_1,35.8_ = 0.30	F_1,47_ = 0.05	F_1,46_ = 2.84	F_1,45_ = 0.35	F_1,43.9_ = 0.07
	0.055 ± 0.015			−0.03 ± 0.02		
Session * ticks	F_1,26_ = 2.0	F_1,37.4_ = 6.85 *	F_1,48_ = 0.58	F_1,46_ = 5.62 *	F_1,46_ = 2.41	F_1,46_ = 0.46
		1.5 ± 0.6 E^−3^		−1.1 ± 0.4 E^−3^		
Session * ticks * *Borrelia*	F_1,24.9_ = 0.25	F_1,34.5_ = 0.36	F_1,46_ = 2.41	F_1,46_ = 4.9 *	F_1,44_ = 0.45	F_1,44_ = 3.91
				1.6 ± 0.7 E^−3^		
Random						
Bird individual	± 0.4 E^−3^	1.0 ± 1.1 E^−4^	0.0 ± 0.0	0.0 ± 0.0	2.1 ± 0.0 E^−17^	0.0 ± 0.0
Residual	2.5 ± 0.7 E^−4^	3.7 ± 1.1 E^−4^	4.5 ± 0.9 E^−3^	2.2 ± 0.5 E^−5^	2.2 ± 0.5	1.0 ± 0.2

Summary of the Type III - tests of the generalized linear mixed models of the change in the great tit’s physiological parameters (‘Δ Cumulative’, Fig. 1) in dependence of the *Borrelia* infection rate in the ticks and the total number of ticks that fed on the birds. Reported estimates and significance levels of the non-significant fixed effects are those before final exclusion in the stepwise selection. Statistics and estimates for the final model are put in bold.

***, **, *, ‘ ’: *P*-value respectively <0.001; <0.01; <0.05; >0.05.

**Table 2:** Changes in acute effects

	Health measures		Immune measures			
	Body condition	Haematocrit	Sed. Rate	Nitric Oxide	Granulocyte	Agranulocyte
Fixed						
Δ Inf.	F_1,47_ = 0.33	F_1,48_ = 13.01***	F_1,47_ = 0.53	F_1,43.2_ = 3.66	F_1,43.9_ = 4.46	F_1,21.1_ = 1.41
		−0.0012 ± 0.0003	0.001 ± 0.001	0.2 ± 0.8 E^−4^		
Δ Inf. * session	F_1,46_ = 0.05	F_1,47_ = 3.46	F_1,47_ = 9.09 **	F_1,38_ = 11.77 **	F_1,25.3_ = 4.32	F_1,26.9_ = 0.51
			−0.003 ± 0.001	6.7 ± 1.9 E^−4^		
Δ Inf. * session *Borrelia	F_1,44_ = 0.15	F_1,45_ = 0.02	F_1,45_ = 0.01	F_1,39.3_ = 12.32 **	F_1,39.3_ = 0.06	F_1,44_ = 0.89
				−0.0010 ± 0.0002		
Δ Inf. * session * ticks	F_1,45_ = 0.50	F_1,46_ = 3.34	F_1,46_ = 1.72	F_1,38.1_ = 0.16	F_1,41.4_ = 1.02	F_1,45_ = 2.1
Δ Inf. * session * ticks * *Borrelia*	F_1,43_ = 0.08	F_1,43.7_ = 0.91	F_1,44_ = 1.74	F_1,38.3_ = 1.1	F_1,38.7_ = 0.06	F_1,43_ = 0.26
Random						
Bird individual	0.0 ± 0.0	0.0 ± 0.0	0.0 ± 0.0	1.1 ± 0.7 E^−5^	2.1 ± 1.4	0.1 ± 1.6
Residual	8.9 ± 1.8 E^−4^	8.9 ± 1.8 E^−4^	10.4 ± 2.0 E^−3^	2.1 ± 0.6 E^−5^	4.3 ± 1.3	7.9 ± 2.3

Summary of the Type III - tests of the generalized linear mixed models of the change in acute tick effects (‘Δ Acute’, Fig. 1) on the great tits’ physiological parameters (Δ Inf.) in dependence of the *Borrelia* infection rate in the ticks and the total number of ticks that fed on the birds. Reported estimates and significance levels of the non-significant fixed effects are those before final exclusion in the stepwise selection. Statistics and estimates of the final model are set in bold.

***, **, *, ‘ ’: *P*-value respectively <0.001; <0.01; <0.05; >0.05.
